# Medical student perceptions of autism education: A qualitative study

**DOI:** 10.3389/fresc.2023.1096117

**Published:** 2023-02-28

**Authors:** Laura Gallaher, Ceri Butler, Sube Banerjee, Juliet Wright, Ann White, Stephanie Daley

**Affiliations:** ^1^ Centre for Dementia Studies, Brighton and Sussex Medical School, Brighton, United Kingdom; ^2^ Department of Medical Education, Brighton and Sussex Medical School, Brighton, United Kingdom; ^3^ Department of Community Paediatrics, Sussex Community NHS Trust, Brighton, United Kingdom

**Keywords:** autism, medical student, education, awareness, workforce

## Abstract

**Background:**

The global prevalence of autism is reported to be at least 1% and is rising. Autistic people have a range of comorbidities resulting in a high use of health services. Doctors of nearly all specialties are likely to encounter autistic people in their practice. Autistic people report dissatisfactory care and encounter disproportionately worse health-related outcomes than non-autistic people, which in part has been attributed to a lack of skill and awareness in the medical workforce. At present, autism education is not always included in undergraduate medical curricula. In England, the Department of Health and Social Care has mandated that autism education should be included in all undergraduate medical curricula but current evidence relating to the delivery and receipt of autism education is poor. A greater understanding of medical student perceptions of autism education is required to inform curriculum development. This qualitative study sought to explore the perceptions of autism education in final year medical students at a medical school in South-East England by 1) assessing their perceived preparedness to care for autistic people once they have graduated from medical school and 2) determining their perceived acceptability of a new undergraduate education programme, Time for Autism (TfA).

**Materials and methods:**

A purposeful sample of ten final-year medical students were recruited. Students completed in-depth, individual interviews. Data was analysed using thematic analysis.

**Results:**

Four key themes were identified: Learning environment, Exposure, Relevance and Curricular priority. The findings of this study indicate that medical students perceived greatest value in autism education when it was directly relevant to developing preparedness for practice. Value was influenced by the perceived curricular priority attached to autism education. The new autism programme, Time for Autism was perceived to add relevance and priority to autism education in the existing curriculum in this medical school setting.

**Discussion:**

The study findings shed new light on medical education literature, emphasising the importance of congruence between the provision of autism education and the prioritisation of autism education within the curriculum. Consideration of relevance and curricular priority can be used to support the development of autism education in future medical curricula.

## Introduction

The rate of autism diagnosis is increasing globally (1). In the United Kingdom, prevalence is currently estimated to be 1%–2%, with approximately 1 in 100 children and 2 in 100 adults diagnosed with autism (2). Autistic people are more likely to experience comorbidities, leading to high service use across a wide range of areas of medicine (3). Collectively, this implies that doctors of all specialties are increasingly likely to encounter autistic people in their practice.

Autism is characterised by impairments of social interaction, language and communication skills and atypical, repetitive behaviours and interests (1). Doctors need to be equipped with the knowledge and skills required to: recognise the signs and symptoms of autism; initiate appropriate diagnostic pathways and; provide good quality care to autistic people.

In the United Kingdom, the average time between the initial presentation and autism diagnosis is 3.6 years in children and approximately 5 years for adults (4). In children, this delay reduces the demonstrable cognitive, linguistic and behavioural benefits of early intervention, compounds parental stress and delays the adoption of effective coping strategies within families (3, 5). Furthermore, early intervention has been shown to ameliorate core symptoms of autism, which are associated with premature mortality (6). Timely diagnosis relies upon doctors having the knowledge and skills to recognise the diverse signs and symptoms and varying presentations of the autistic spectrum.

The core features of autism often manifest as being unable to tolerate change and feeling threatened by unfamiliar people and environments. The associated anxiety, sensory overload and distress significantly impact the patient experience (7). In the United Kingdom, the Equality Act requires doctors to make reasonable adjustments for autistic people in order to support their needs (8). Autistic people and their caregivers value this flexible responsiveness as a key determinant in patient satisfaction (9).

Despite this need, doctors report a lack of understanding about autism (10). This lack of understanding could negatively impact the quality of life of autistic people and lead to serious consequences in terms of health-related outcomes. Reports of dissatisfactory care (11–13), disproportionately worse health-related outcomes (14) and premature mortality (6) have all been linked to concerns relating to a lack of skill and knowledge in the medical workforce.

In response to these concerns, a new legislative requirement in England has been introduced to ensure that the health and social care workforce undertake autism (and learning disability) training appropriate to their specific role (15), with a supporting skills framework for caring for autistic people (16). Within this new legislative requirement, this includes the future workforce, namely medical students, however, it is not clear as to how medical students are best taught about autism.

In the United Kingdom, medical student knowledge about autism (17) and perceived preparedness to care for autistic people (18) are both reported to be low. Autism education for medical students is described to be sparse and, in many cases, absent entirely (19). The paucity of autism education in the training of doctors represents a missed opportunity for developing competency in this area.

A medical school in the South of England recognised the need for enhancing autism education and a new educational programme called Time for Autism (TfA) has been developed. Within Time for Autism, pairs of medical students will be partnered with a family with a school-aged autistic child. Medical students will visit the family three times over a period of 1 year, during the fourth year of their medical training. Students will be encouraged to engage with the family to learn from their experiences, to understand the challenges autistic people (and their families) encounter and to explore how doctors can best support them. Based on clinical experience, it is assumed that between 15%–20% of parents taking part in TfA will also be autistic, meaning the programme is not specifically focused on autistic children. TfA was based on the Time for Dementia model of education, which demonstrated that longitudinal contact between medical students and those living with dementia had a positive impact on student knowledge and attitude (20, 21). To support the development of TfA, this qualitative study was undertaken. The aim of the study was to gain an understanding of medical student's perceptions of autism education and the acceptability of the proposed autism education programme, TfA.

## Method and materials

### Design

A qualitative study was undertaken to understand the *reality* of participants' perceptions through an exploration of their experiences and the interpretations they attached to them, within a broader *social construct*. This contextualist epistemological approach (a bridge between realism and constructivism) (22) loaned itself to the phenomenological methodology of thematic analysis and is steered by Braun and Clarke's guided approach to thematic analysis (23).

### Sample and setting

A target of a purposive sample of 20 final year medical students from a medical school in the south of England was sought. All final year medical students (*n* = 132) were eligible to participate, not just those students planning to become paediatricians. All students had received one hour of autism teaching in the classroom and a further two hours of teaching during their paediatric rotation, as well as ad-hoc opportunities to meet with autistic children and adults during rotations.

### Procedure

First, all final year medical students were emailed with a brief introduction of the study, and a study information sheet. Second, a researcher (LG) attended a teaching session, which 92 students attended, and all students were invited to give consent to be contacted about taking part in the study. No incentives for participation were offered. 57 students provided written consent to be contacted about the study. Third, all 57 students were invited to take part in a qualitative interview by email, and 16 responded positively. However only 10 students responded to possible interview times. Fourth, at the beginning of each interview, written informed consent was obtained.

A topic guide was used to support the interviews. The topic guide was developed from a review of the literature on autism teaching, and is provided as a Supplementary File. Four topic areas were covered: student's previous experiences of autism; perceptions of autism teaching during their medical training; perceived preparedness to care for autistic people at the point of graduation from medical school and; perceived acceptability of TfA. A series of prompts were included to explore each area further. Students were asked, in order, to critique how autism was included in their undergraduate curriculum; to discuss their perceived preparedness to treat autistic patients and; to discuss their perceived acceptability of the TfA model. Any comparisons drawn or preferences stated were provided by the participants independently.

Interviews were conducted by LG, who was unknown to the participants. LG had undertaken post-graduate training on qualitative research methods and was supervised by two qualitative researchers (SD and CB). Interviews took place at times and venues convenient to the participant. Nine interviews were conducted in medical school buildings and one interview was conducted by telephone, all during normal working hours. All interviews were audio-recorded and transcribed verbatim and checked for accuracy by the researcher.

The target of 20 students was not met, and only ten students participated in in-depth individual interviews. On average, interviews lasted 27 min and ranged between 18 and 47 min in duration. The interviews took place between February and April 2019.

### Analysis

Interview data were analysed inductively using thematic analysis practices described by Braun and Clarke (23). The first two interview transcripts were reviewed independently by two researchers (LG and SD), who assigned descriptive codes to meaningful sections of text and then met to discuss and agree the list of potential codes. This was refined by coding one further transcript and a coding framework was developed, which was subsequently applied to the remaining transcripts. The researchers met regularly to discuss, refine, separate and discard themes, and to identify relationships between the themes. The thematic analysis was supported by NVivo 12 (QSR International) software, which was used to highlight meaningful portions of text and to categorise data into themes. Thematic saturation was determined through the consistency of repeated themes within transcripts and the paucity of newly emerging themes in the last four transcripts analysed.

### Ethical considerations

This study was approved by the Health Research Authority, Health and Care Research (Wales) (Ref: 19/SC/0041). Students were provided with full details of the study and advised that participation was voluntary. Confidentiality was maintained through processes of data anonymisation and redaction of identifiable information from transcripts. Original recordings were destroyed following transcription. There was a small risk that students could become upset during interviews. If students became upset, it was agreed that they would be asked if they wanted to terminate the interview and be signposted to relevant university support services.

## Results

Ten medical students completed in-depth individual research interviews. The characteristics of participants are summarised in Table 1.

**Table 1:** Characteristics of study participants.

Total Number of students	Study sample	Entire student year group
	10	156
Characteristic	Median	Median
Age (years)	23.5 (range = 22–34)	24 (range 22–50)
	Number (%)	No (%)
**Gender**		
Male	4 (40)	64 (41)
Female	6 (60)	92 (59)
**Ethnicity**		
White British/European	7 (70)	103 (66)
Asian/Asian British	1 (10)	26 (16)
Mixed/multiple ethnic groups	1 (10)	7 (5)
Black/African/Caribbean/Black British	0 (0)	6 (4)
Other	1 (10)	5 (3)
**Prefer not to state**	–	9 (6)
**Personal experience of autism**		
Yes	4 (40)	–*
No	6 (60)	–
**Details of personal experience**		
Had been assessed for autism	1 (10)	–
Autistic family member	1 (10)	–
Vocational experience working with autistic people	1 (10)	–
Autistic family member and vocational experience with autistic people	1 (10)	–

* not known.

Sociodemographic characteristics of age, gender and ethnicity were broadly representative of the final year cohort, but participants varied in their experiences of autism outside of their curriculum. One student had been assessed for autism. Two students had autistic siblings, one of whom also had vocational experience working with autistic people. One further student also had vocational experience working with autism.

Four overarching themes were derived from the analysis. These were Learning environment, Exposure, Relevance and Curricular priority.

### Theme 1: Learning environment

Students provided a detailed critique of the perceived value of three distinct learning environments: classroom-based didactic teaching, learning in a clinical setting, and meeting autistic people in the community. Students reflected on didactic and clinical experiences within their medical education. They drew upon their experience of undertaking a longitudinal dementia programme: Time for Dementia, to consider the perceived learning value of meeting autistic people and their families in the community.

### Didactic teaching

Students had limited recall of a child development lecture which provided teaching about autism in the pre-clinical phase of their course. Students reflected that more focussed and dedicated didactic teaching during the clinical phase of their course would have been useful in providing a theoretical foundation to supplement encounters with autistic people in that phase of their training.

*“I could be wrong, but I don’t remember having a specific kind of formal lecture.”*

*Student 1*

### Clinical setting

All of the students reflected on ad-hoc opportunities through their rotations to attend neurodevelopmental, GP specialist and adult autism clinics. Students who attended neurodevelopmental clinics appreciated the opportunity to observe an autism assessment, including observing a child's behaviour, the different components of the assessment procedure and multidisciplinary teamwork. Collectively, this helped students understand the complexity of autism assessments and which traits were important for diagnosis.

*“…it just sticks in your head more, because you’re doing it and having to think about how to.”*

*Student 9*

The value students placed on ad-hoc learning opportunities in clinic related to their level of involvement during the experience. For example, several students were asked to play with the child during the neurodevelopmental clinic. Some students felt that this precluded the opportunity to learn from the interaction between the parent and the clinician, whereas those students who were provided with an opportunity to discuss their observations with clinicians, perceived practical learning benefit from the experience.

*“I ended up almost minding the child a bit, while the paediatrician spoke to the mother, which was a bit of a shame in a way, because I kind of missed the actual nitty-gritty of the assessment… it would have been a bit more useful if I’d actually been there in the assessment and looking at the kind of questions they were asking the mum”*

*Student 1*

Whilst students described the aforementioned experience as an opportunity to observe the clinician's communication skills and adjustments to practice with autistic people, it was also recognised that it wasn’t a standardised learning opportunity for all students.

*“I think they tried to get people into various neuro-developmental clinics, but I’m not sure everyone gets a chance to actually see patients.”*

*Student 2*

For students who lacked personal experience of autism, placement exposure wasn't sufficient to enable them to suggest any adjustments which might support the care of autistic people beyond the use of simple and literal language. Students were unclear how they might translate these adjustments into their own professional practice and questioned their preparedness.

*“it's helped me in that way, but obviously I’ve not been like a proper doctor yet, on the wards, so how easy all of that will be to do…?”*

*Student 3*

Students identified the broader challenges of learning in all clinical settings when a clinician might need to prioritise patient care over teaching students. This was particularly the case for some students who observed an autism assessment, but where the clinic appointment concluded before a diagnosis had been made. This left the students unclear of whether their own observations were consistent with, or sufficient for, a diagnosis of autism, with no opportunity to discuss their observations.

*“I went to a Child Development clinic, so yes, but again it was at the very infancy of a child getting a diagnosis of autism. So, I don’t know what the actual final outcome was,”*

*Student 8*

### Community setting

Students questioned the value of the clinic setting for observation-based learning, reflecting that the duration and nature of the clinic environment might not provide opportunity to truly understand autistic behaviours, and applauded the proposal, that TfA would include opportunities to meet autistic people in the community.

*“I think definitely having an understanding of how autism impacts on the patient's life and the family life as well, for instance, maybe the impact on other siblings and parents. Maybe learning a bit more in depth about the specific domains that people with autism have difficulty with, and it might make me a little bit more aware of it when I’m practising in the future. So, you probably learn some tips as well from the parents who know quite well, and have probably come up with really good ways of communicating and ways of helping their children, and I think those tips would be really useful for us to have, especially, yeah, I mean whatever we go into, really, GP, paediatrics, it would be really useful to have that.”*

*Student 1*

*“I mean the proposed Time for Autism I think is a really good idea, because it worked with dementia.”*

*Student 5*

*“Well, that's the thing, I always received Time for Dementia quite well, I thought it was a good idea and I think I did benefit from it. So, I personally would be okay with Time for Autism.”*

*Student 2*

Students considered the perceived learning benefits of meeting families in various community settings, including the family home, the child's school, a play/support group and a playground, alongside expressing concern for the autistic child.. Most students supported visiting families in multiple locations, as a way of providing opportunity to observe different behaviours.

*“if you got to see the family maybe once at home, and then once at school and once in a completely different environment, then that would actually be quite good and broad to see how the child adapts in different environments”*

*Student 8*

Whilst students positively appraised the perceived added value of meeting autistic people in the community, concerns were expressed about the additional time burden, travelling, additional academic demands and the repetition of visits.

“*I think time, so having to travel very long distances, or having to do a very large number of visits, especially in your clinical years I think would add a certain level of stress, just because you already have so much to do, studying wise”*

* Student 1*

“*Going back to what I said about Time for Dementia, perhaps that overkill was it was the same conversations in the same place, doing the same things, eating the same tea and biscuits*

*Student 7*

### Theme 2: exposure

All students agreed that exposure to autistic individuals provided a more valuable learning experience than theory-based learning.

*“I think it would make me feel more prepared, definitely more prepared… because, I mean, you can know the facts, you can know what to look out for but unless you see it… I need to see it, I need to experience to really learn from it.”*

*Student 9*

*“I think I would have been better prepared with a programme like this, because it's about exposure.”*

*Student 5*

The value placed on exposure to autistic people over theory-based learning was associated with learning from lived experience, longitudinal contact and breadth of exposure.

### Learning from lived experience

Students drew from their experience of the Time for Dementia programme and perceived that learning from lived experience might help to understand what it is like to live with autism and the challenges that it poses.

*“…just kind of be a bit more empathetic, I think and be a bit more understanding and know that there's not just… autism is not just a diagnosis but a range of things and it affects the patient and their family's whole life. So, keep a lot of those things in mind when speaking to someone with autism.”*

*Student 6*

Furthermore, most students perceived that increased exposure to families with an autistic child could aid the development of communication skills and help them feel more confident in interacting with autistic people, post-graduation from medical school.

*“…you probably learn some tips as well from the parents who …have probably come up with really good ways of communicating and ways of helping their children, and I think those tips would be really useful for us to have.”*

*Student 1*

### Breadth of exposure

Most students positively rated the opportunity for relational learning and saw benefit in learning through longitudinal contact, as proposed in the TfA programme.

*“it would be good to, you know, just follow up, see what are good days, see what are bad days, see how it can impact people's lives. You don’t get anything like that from a clinic.”*

*Student 5*

However, some students were concerned about how much could be learned from repeated exposure to a single family and perceived that it limited the opportunity to learn about the heterogeneity of the autistic spectrum. Students offered various mitigations to overcome this perceived limitation of TfA, including visiting more families but less frequently or ensuring additional opportunities for exposure to autism elsewhere in the curriculum. Others were more open to sharing reflective experiences and learning from peers.

*“I know that would be probably quite logistically difficult, but I suspect, you know, the problem with just seeing one person is that everyone's different with autism… I guess that would be in the sharing of experiences between people, and whether that should be in a formalised manner in terms of like study groups or group reflection exercises.”*

*Student 2*

*“I think that would be really useful, maybe just having a panel of adults with autism, but not the same type of presentation… I think the students would go away understanding autism a lot more.”*

*Student 4*

### Theme 3: relevance

All students acknowledged that they were likely to encounter autistic people in their practice, regardless of their chosen specialty. Students felt that education needed to be directly relevant to preparing for practice, highlighting the perceived importance of learning in a context that corresponds to training in the first two years after medical school graduation, emphasising the need to develop autism-specific skills required for practice in the immediate period post-graduation.

*“I don’t necessarily think it should be focused on the sort of medical aspect in terms of kind of diagnosis and management… as a very junior doctor, you know, that's not going to be our job, making a diagnosis, and I think that's, you know, it's a thing with all our exams really, that, you know, the exams don’t necessarily represent what we’re going to be doing as a job. But I mean we can’t fix that really… the time when I’m likely to see it is going to be working, you know, in kind of an in-patient setting, either in the emergency department, or on wards… And autism will be completely different in those situations than it would be in the sort of calm settings that I’ve seen it so far.”*

*Student 10*

Students recognised the importance of both hard and soft skills to prepare for practice, and an awareness that these skills are learned in different ways and require different modes of delivery in the curriculum. Students perceived that development of hard skills, such as clinical knowledge, was associated with didactic teaching in the context of parallel exposure to autism. The development of soft skills, such as empathy and communication skills, was associated with interaction with autistic people. Observation was not deemed sufficient for the development of soft skills.

*“I do know that some people find it incredibly awkward to talk to children and maybe the autism on top of that, they don’t want to say the wrong thing, or do the wrong thing, or cause any distress. And so that may make some students feel quite uncomfortable, but that's the very point of the programme, I imagine, is to you know, expose people to that and normalise and then have them prepared for that”*

*Student 5*

Students deliberated the position of autism education in the medical undergraduate curriculum and agreed that a programme that includes exposure to autistic children, such as TfA, would feel most relevant alongside their paediatric rotation, to complement learning about child development. However, students were concerned about the additional burden it might pose in an already busy clinical phase of their training.

*“Yeah, so from that point of view it would work quite nicely with the year, we get assessed on paediatrics, so I think it would be received well.”*

*Student 8*

Most students emphasised the perceived importance of autism education having clearly defined learning outcomes that link directly to caring for autistic people. Despite concerns relating to additional burden in the curriculum, students assigned value to reflection. Students considered that reflection would serve multiple purposes including being able to contemplate, as well as demonstrate, learning in the context of perceived preparedness.

*“I think reflection's good because it gets the student to think “What did I learn from this? What did I get from this?”*

*Student 5*

### Theme 4: curricular priority

Students perceived that a curriculum which does not guarantee autism learning opportunities or which only includes non-mandatory, non-standardised learning opportunities gives the impression that autism is not an important condition to learn about, leading to poor student engagement.

*“It doesn’t feel like it's, you know, a serious topic because ‘oh, we’ll pop it in the side here’”*

*Student 4*

### Standardised learning

Despite reservations about non-standardised learning, students recognised the value of authentic learning experiences which are, in most instances, non-standardised and cannot guarantee the opportunity for all students. Students suggested the inclusion of supplementary standardised learning experience as a means of ensuring learning and enhancing the perceived necessity for competency in understanding autism.

*“*[the medical school] *have to cram in so many different medical conditions* (to teach to student)*, but if they were going to do it* (teach autism)*, I think there needs to be, if this is it, then maybe a conference or maybe a video or a lecture that's* *standardised* (all students to receive the same autism teaching)*, that every kind of student gets…,.”*

*Student 3*

*“I feel very lucky to have been to that clinic that I described, I’m not sure many people have been there, as really the only exposure I’ve had.”*

* Student 7*

### Learning outcomes

Without sufficient perceived emphasis in the current curriculum, some students felt unclear about what competencies were required to care for autistic people and what the learning outcomes of autism education were.

*“I think the learning, or sort of the outcomes that students need to achieve should be quite clear and targeted at the sorts of things that we’re likely to encounter working as a very junior doctor in an acute hospital.”*

*Student 10*

Most students described themselves as having good, and transferable, communication skills and added that they would be able to defer to a caregiver if they had any difficulty communicating with an autistic person. These students felt sufficiently prepared to care for autistic people. A small number of students were less certain about their competency and described a lack of preparedness.

*“I think I’ve got the transferable skills, communication skills, mental health knowledge to deal with that. But at the other end of the* [autistic] *spectrum, no, not well prepared at all. I wouldn’t be well prepared for that at all.”*

*Student 7*

### Assessment

Students expressed a significant exam-focus, sharing that they prioritised components of the curriculum that were assessed. They promoted the use of formal assessment as a way of encouraging students to engage in autism education.

*“…so let's say if it was a formalised thing where you had to… you know, you got a mark for it and it accounted for the whole year, it went towards your decile score for example, I think people would take it a lot more seriously.”*

*Student 2*

## Discussion

This study sought to gain an understanding of medical students' perceptions of autism education. Students reflected on autism learning experiences within their undergraduate curriculum, recognising limitations in their knowledge and understanding. Despite describing incompetence, students presented mixed views about their perceived preparedness to care for autistic people. Students perceived that the TfA programme would be broadly acceptable to students and would provide additional valuable opportunities for learning from lived experience but expressed concerns relating to burden and breadth of exposure.

The findings of this study provide valuable insights into how students perceive autism education. Students placed most value to learning experiences that prepare them for practice and perceived that student engagement depends upon the perceived *relevance* of autism education to future practice and the perceived *curricular priority* with which autism education is included in the curriculum.

Students expressed the need for experiential learning in a context relevant to training in the first two years after medical school graduation, to facilitate preparedness. This included the development of harder skills within a clinical setting and softer skills when provided the opportunity to learning from lived experience. This adds to existing literature about the relationship between availability of contextualised experiential learning opportunities and preparedness (18, 24) and mirrors well-established learning theories, such as Kolb's experiential learning cycle, which imparts that learning facts and ‘seeing’ is not sufficient and that learning requires ‘doing’ and active reflection (25).

This presented a problem in the current curriculum: students understood their learning needs in relation to preparedness but did not perceive that the autism education within their curriculum was appropriate to meet them. This awareness contributed to the judgements that students made about the priority with which autism education was included in their curriculum.

Perceived curricular priority was associated with a mandatory autism education that includes both didactic teaching and guaranteed exposure to autistic people, as well as formalised assessment of clearly defined learning objectives. Perceived curricular priority was negated if the opportunity to develop hard skills was non-standardised and if exposure to autistic people did not guarantee an opportunity to develop soft skills (and hard skills to a lesser extent) through experiential learning. Perceived curricular priority was further negated if the development of such skills was not scrutinised by student assessment.

Including autism education without demonstrable curricular priority presented a paradox for students. Students sensed that they were being offered elective opportunities for autism education, without any clear understanding of why, leading to poor student engagement. This conundrum exemplifies the power of the ‘hidden curriculum’ (26). A hidden curriculum that contradicts the formal curriculum risks students misinterpreting the priority of autism education and discourages student engagement, which interferes with development of preparedness.

Despite students asserting that they had not received sufficient relevant autism education, most students perceived that transferable communication skills would be sufficient for preparedness. This incongruence suggests that lack of curricular priority prevented students acquiring an awareness of, or an understanding of the importance of, the competencies required to care for autistic people. Students were unconsciously incompetent, unaware of their lack of preparedness.

**Figure 1:**
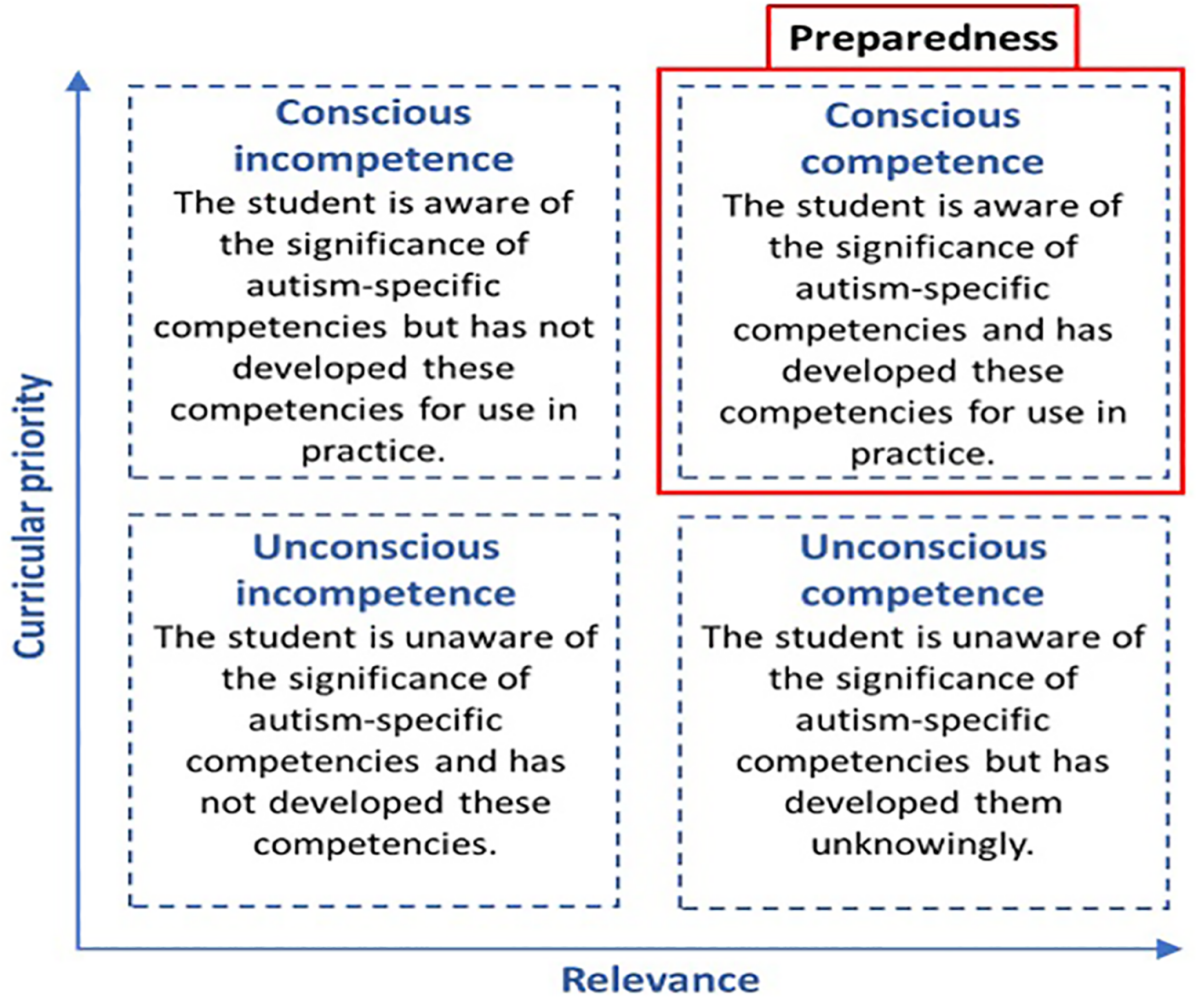
The contribution of curricular priority (conscious-provoking) and relevant (competency-driven) autism education of the development of preparedness.

The interplay between the provision of relevant autism education and perceived curricular priority, and the subsequent development of preparedness can be mapped onto Maslow's Conscious Competence model (27), as shown in Figure 1.

In this revised construct, preparedness is described as conscious competence and is the sum of: 1) a formal curriculum that includes autism education that is competency-driven (relevant) and; 2) hidden and formal curricula that provoke conscious awareness of autism-specific competencies (curricular priority). This model illustrates the importance of congruence between the formal and hidden curriculum in the development of preparedness.

Students described that they engage most with education they perceive to be relevant and prioritised within the curriculum. A curriculum that provides autism education in this way has the greatest potential to engage students, develop their preparedness and improve health-related outcomes for autistic people.

Students perceived TfA to be a broadly acceptable education programme that provides opportunity to improve preparedness through delivery of *relevant* autism education. The relevance attributed to TfA was associated with experiential opportunities perceived to extend knowledge acquired through didactic teaching; develop and apply skills through interacting with autistic people and develop a deeper understanding of the impact of autism on an individual and their family. Some students perceived that in-depth longitudinal learning with the same family might compromise the breadth of autism exposure. To address this concern, in TfA, medical students will undertake three visits to a family with an autistic child, as well as attending a wider stakeholder conference where they will meet other families with an autistic child. Students also perceived that TfA added *priority* to autism education through provision of mandatory autism education. However, TfA adds to an already busy curriculum, and students encouraged the use of assessment to enhance student engagement, which has been incorporated into the programme.

This study rationalises the following recommendations for TfA and for undergraduate medical curriculum design more broadly. First that the curricula should provide autism education that is directly relevant to practice, immediately post-graduation from medical school. Second, that curriculum design should emphasise autism education in such a way that the priority identified at a national level is perceived by students, encouraging motivation to engage. Within England, it is possible that the statutory requirements for autism training will bring pressure to medical schools to prioritise autism education. Future research which seeks to survey medical schools to ascertain curriculum priority (through teaching delivery and assessment) to review progress should be undertaken, as has been carried out in other neglected areas of teaching such as dementia (28) and learning disability (29).

### Strengths and limitations

The intention of this study was to understand medical student experiences and views of routine autism education to support the development of a new autism programme, TfA. We are not aware of any other studies which have sought to understand medical student experiences and views about routine autism education. The findings from this study, along with a concurrent study on the views of parents of autistic children about medical care and TfA (30), allow for lived experience to be fully incorporated within the TfA programme. The findings also offer value to other educators.

The study was designed to optimise rigor in accordance with four elements of the trustworthiness criteria for qualitative research (31); namely credibility, transferability, dependability and confirmability, This was achieved as follows:

Credibility: Choice of research methods, independent researcher, confidentiality
Transferability: Focus on the general experience of autism teaching for medical students. Level/(traditional) approach to teaching likely to be similar to other medical schools, and therefore of value to other educators.
Dependability: Joint data analysis between two of research team, the use of reflexivity, Audio recording of interviews.
Confirmability: Audio recording of interviews, use of topic guide and Nvivo software.

This study has four limitations. The first and main limitation of this study relates to the potential for sampling bias. The original research proposal sought to complete 20 individual in-depth interviews, with a view to being sufficient to attain thematic saturation. Despite 57 students consenting to being contacted, only 16 responded and 6 of them were unable to commit to a time to meet before leaving the area to go on their final medical elective. Thematic saturation was achieved but the sample might not have been representative of the study population by including students with a special interest in autism/strong feelings about the curriculum, as it is very unlikely that 40% of the entire student year group had experience of autism. Despite these limitations, we believe that our findings are of value, given the need for improved autism education and the lack of evidence in this area. The findings shine a light on the student experience of routine autism teaching (didactic lectures and opportunistic placements) which are very likely to be a common teaching experience in many medical schools.

Second, students were only asked to consider the perceived value of autism education in their current education and in the proposed TfA model. This limits any conclusions that can be drawn from different models of delivery for autism education. Whilst the evidence of the effectiveness of autism teaching for medical students is limited, using a panel of professionals and parents along with lectures has been found to improve levels of self-reported knowledge, skills, confidence and comfort in working with autistic people (32), as well as the use of video materials of lived experience to support autism teaching (33, 34). For the existing workforce, the ECHO model of remote ‘clinics’, which provides an interdisciplinary expert team including brief didactic teaching, case studies and guided practice, has demonstrated changes in practice following participation (35). Presenting any of these evidence-based models may also have been acceptable to participants. It is also possible that more focussed use of ‘real life’ learning in rotation settings could have been acceptable for students, although this raises the significant challenge of standardisation and difficulty in ensuring guaranteed clinical contact.

Third, interviews were brief as they only focussed on autism education, with the mean average of interviews being 24 min for those without experience of autism, and 29 min with those with lived experience. Therefore, the broader context of the experiences of students and their perceptions of their medical education, which might have added to the interviews, was not explored.

Fourth, the final limitation is that the research only took place in one medical school. Further research is indicated to explore medical student perceptions in different medical schools in order to explore the transferability of findings.

In conclusion, this study sought to explore final year medical student perceptions of autism education. Final year medical student perceptions of autism education are intrinsically linked to the perceived relevance of autism education to future practice and the perceived priority with which it is included in the curriculum. This study advocates student-centred learning and highlights the value of involving students in pedagogic consultation.

## Data Availability

The raw data supporting the conclusions of this article will be made available by the authors, without undue reservation.
