# Supplementary material for: Medical student perceptions of autism education: A qualitative study
Source: Front Rehabil Sci. 2023 Feb 28;4:1096117. doi: 10.3389/fresc.2023.1096117 (PMC10011116; doi:10.3389/fresc.2023.1096117)
Supplement: Supplementary file 1 [file Datasheet1.pdf]

Time for Autism

Study Topic guide

Introduction *(prompts: consent, background to the study, plan for interview)*

Topic area to explore:

Area one:

What has been your experience of Autism?
(prompts:, Clinical experiences, Personal experiences)

Area two:

Can you tell us about the Autism education which you have received?
(prompts: Lectures, Paediatrics rotation, Elective modules)

Do you feel that your medical education in this area has been sufficient?

Area three:

What are your thoughts about treating patients with Autism when you qualify?
(prompts: thoughts, worries, concerns, medical role)

Do you feel confident about the following:
identifying signs and symptoms /diagnosing Autism;
support you can offer (i) patients (ii) parents/carers,
sources of extra information/support)

Area four:

Views about Time for Autism (TfA)
(prompts: description of programme, thoughts/feelings/reservations about visiting TfA families, how would TfA have enhanced your learning? anticipated issues in delivery/acceptability?)

Anything else
